# Exploring the characteristics of patients with mesothelioma who chose active symptom control over chemotherapy as first-line treatment: a prospective, observational, single centre study

**DOI:** 10.1186/s12904-017-0255-3

**Published:** 2017-12-08

**Authors:** Anna C. Bibby, Duneesha De Fonseka, Anna J. Morley, Emma Keenan, Alfredo Addeo, Sarah Smith, Anthony J. Edey, Nick A. Maskell

**Affiliations:** 1 Academic Respiratory Unit, School of Clinical Sciences, University of Bristol, Southmead Hospital, 2nd Floor L&R Building, Bristol, BS10 5NB UK; 2 Department of Respiratory Medicine, North Bristol NHS Trust, Southmead Hospital, Bristol, BS10 5NB UK; 3 Bristol Cancer Institute, Bristol Haematology & Oncology Centre, Horfield Rd, Bristol, BS2 8ED UK; 4 Department of Radiology, North Bristol NHS Trust, Southmead Hospital, Bristol, BS10 5NB UK

**Keywords:** Mesothelioma, Active symptom control, Chemotherapy, Treatment decisions, Best supportive care

## Abstract

**Background:**

Mesothelioma is an aggressive thoracic tumour with a poor prognosis. The only treatment that extends survival is chemotherapy. However, in the UK, up to 50% of patients who are suitable for chemotherapy choose not to receive it, opting for active symptom control instead.

The aim of this prospective, single-centre observational study was to describe the characteristics of patients who chose active symptom control over chemotherapy and explore their reasons for doing so.

**Methods:**

Two hundred consecutive patients with mesothelioma from one UK centre were included. Eligibility for chemotherapy and choice of first-line treatment were recorded prospectively. Patient characteristics and outcomes were compared using descriptive statistics, regression analysis and survival analysis. Reasons for choosing active symptom control over chemotherapy were extracted, retrospectively.

**Results:**

People who chose active symptom control were older, more likely to be female and had worse performance statuses than patients who received front-line chemotherapy. Concern over side effects, the modest survival benefit and previous adverse experiences with chemotherapy were reported as reasons for the decision.

Median survival was 13.9 months in the chemotherapy group compared with 6.7 months in the active symptom control group.

**Conclusions:**

This is the first study to describe the characteristics of patients with mesothelioma who chose active symptom control over chemotherapy, in the front-line setting. Important differences were seen between this group and patients who received chemotherapy, although confounding is likely to have affected some outcomes.

Future research could use qualitative methods to explore patients’ reasons for choosing active symptom control, and to further elucidate the decision-making process.

**Electronic supplementary material:**

The online version of this article (10.1186/s12904-017-0255-3) contains supplementary material, which is available to authorized users.

## Background

Malignant pleural mesothelioma (MPM) is a universally fatal thoracic tumour with limited therapeutic options [1–4]. Chemotherapy is the current standard of care for first-line treatment, although the survival benefits are modest [5–7]. Combination chemotherapy with cisplatin and pemetrexed was the first regimen to demonstrate improved survival in mesothelioma – a phase III randomised controlled trial reported a median survival benefit of 2.8 months with dual therapy compared to cisplatin alone, [5] and a subsequent expanded access program report showed survival enhancements of 4 months [6]. More recently the phase III MAPS trial showed that the addition of bevacizumab to this regimen extended survival by another 2.7 months, although this agent may not be suitable for everyone, and is not universally available [7]. Multiple clinical trials are currently underway investigating novel agents, and it is anticipated that the future of MPM management will include a greater choice of treatment options than is currently available.

Aside from clinical trials, the current alternative to chemotherapy as first-line treatment for MPM is active symptom control (ASC). According to the 2007 British Thoracic Society Statement on Mesothelioma, ASC should include regular specialist follow-up and appropriate symptomatic treatment, such as analgesia, palliative radiotherapy and steroids as required [1]. In a randomised trial comparing mitomycin, vinblastine and cisplatin (MVP) or vinorelbine chemotherapy with ASC, patients who received ASC alone had similar quality of life to patients who received chemotherapy [8]. That same trial, which is the only randomised study to have compared chemotherapy to ASC, also demonstrated no survival difference between the two groups [8]. However, since the trial was undertaken in the pre-pemetrexed era, these results cannot be extrapolated to modern chemotherapy regimens. Whilst the true effect of pemetrexed/cisplatin chemotherapy versus no treatment is currently unknown, it is likely to exceed the 6 months survival benefit conferred by adding pemetrexed and bevacizumab to cisplatin [5, 7]. 

Patients are considered eligible for first-line chemotherapy if they have a WHO performance status (PS) of 0 or 1 and no significant comorbidities [2, 9, 10]. Additionally, some patients who have a PS of 2 may be suitable to receive chemotherapy, based on individual assessment of their physical health [9]. In our centre, patients with MPM are discussed at a regional mesothelioma multidisciplinary team meeting (MDT), where the diagnosis is confirmed and eligibility for chemotherapy determined [11]. Eligible patients are offered first-line chemotherapy by a respiratory physician at their subsequent clinic appointment and given the chance to discuss the benefits and disadvantages of the planned treatment regimen with their treating clinician. Participants who wish to receive chemotherapy, or who wish to discuss the matter further, are referred to an oncologist. Patients who state at the outset that they do not wish to receive chemotherapy are not referred to an oncologist.

A proportion of patients who are offered first-line chemotherapy make an informed decision to receive ASC instead. Epidemiological data collected in Leeds, UK between 2001 and 2005 reported that 28 out of 54 eligible patients (52%) declined chemotherapy [12]. More recently, the UK National Lung Cancer Audit revealed that chemotherapy uptake in MPM patients with PS 0-1 varied from 46% to 71% across UK centres [2]. Given that the majority of these patients would have been eligible to receive front-line chemotherapy, it is likely that a proportion of them made an active decision to receive ASC instead.

Epidemiological data from other countries is difficult to interpret, as performance status and eligibility for chemotherapy is often omitted. However, Kao et al. proposed an optimal chemotherapy utilization rate of 65% based on predictions of eligibility [13]. Chemotherapy usage rates of 36% in the Netherlands between 2005 and 2006, and 54% in Australia between 2007 and 2009 are both lower than this proposed benchmark, again suggesting that a proportion of patients chose not to receive chemotherapy despite being eligible [14, 15]. 

The aim of this study was to explore the characteristics of patients who chose to receive ASC rather than first-line chemotherapy, and to determine what factors were associated with this decision. This information is important, as a treatment is only effective if patients are willing to take it. Similarly, a new treatment may demonstrate encouraging results in clinical trials, but its effectiveness will be reduced if, in real-life, patients chose not to receive it. This is likely to become increasingly pertinent as new treatments emerge for mesothelioma and become adopted into usual clinical care.

Acknowledging there is a cohort of patients who decline first-line chemotherapy, recognising their characteristics, and exploring their reasons for making this choice may help improve treatment uptake in the future. Additionally, this information will afford clinicians and allied health professionals a greater understanding of their patients, and will lead to better communication, particularly in discussions related to treatment decisions and chemotherapy.

## Methods

This was a prospective, observational, single-centre, UK-based study of consecutive patients with MPM enrolled in an ongoing prospective cohort study (Investigating Pleural Disease Study, Research Ethics Committee South West -Central Bristol, ref. 08/H0102/11 – see Appendix 1 for inclusion and exclusion criteria). All patients with a diagnosis of MPM were included, and all diagnoses of MPM were discussed and confirmed at the regional MPM MDT.

Baseline characteristics, symptoms and tumour variables (histological sub-type and IMIG stage [16]) were collected prospectively. Eligibility for first-line chemotherapy was determined at MDT and subsequently confirmed on an individual basis in oncology or respiratory clinics. Patients were considered eligible for front-line chemotherapy if they had a PS of 0 or 1 and no significant organ dysfunction (e.g. cardiac, renal or liver), or if they had a PS of 2 with good physical function and few co-morbidities.

The primary outcome was the proportion of patients who chose to receive ASC having been offered first-line chemotherapy. This decision was recorded prospectively on the study database. Patients’ reasons for choosing ASC were obtained from retrospective interrogation of medical records and clinic letters. Potential reasons for choosing ASC were not defined a priori, as this was considered a hypothesis generating exercise.

The characteristics of patients who were offered first-line chemotherapy and chose ASC were compared with patients who accepted first-line chemotherapy. Chi squared test was used for categorical variables, with Fishers Exact test employed if any individual value was less than 10. Unpaired two-tailed T tests were used for normally-distributed continuous variables and Wilcoxon Rank Sum for non-parametric continuous data. Univariable and multivariable logistic regression was used to explore associations between baseline characteristics and choosing ASC.

The secondary outcome was survival, calculated from date of diagnosis to date of death, censored on 26/06/2017. Survival was calculated for all MPM patients who were offered chemotherapy. Survival in patients who were offered first-line chemotherapy and chose ASC was compared with patients who accepted first-line chemotherapy. Kaplan Meier curves were drawn to visually compare survival between these two groups. Cox Proportional Hazards model was used, with adjustment for age, sex, laterality, PS, histology, stage and symptoms.

Apart from patients’ reasons for choosing ASC, all data were collected prospectively on the study database. Relevant data was extracted from the database by one of the authors (ACB) using a standardised data collection form (shown in Additional file 1: Appendix 2). Patients’ reasons for choosing ASC were obtained retrospectively from patient records.

## Results

Two hundred patients with MPM enrolled in the study between 1/3/08 and 8/6/16, of whom 150/200 (75%) were considered eligible for first-line chemotherapy at initial assessment. 10/150 (6.7%) subsequently became ineligible due to a rapid deterioration in PS between baseline assessment and discussion about chemotherapy. Data were missing on 1 patient who moved out of the area, and was lost to follow up. This person’s data are not included in the analysis.

### Primary outcome

Of 139 patients offered first-line chemotherapy, 93 (66.9%) accepted and 46 (33.1%) chose ASC. The characteristics of these patients are shown in Table 1.

**Table 1:** Characteristics of patients who were offered first-line chemotherapy, whochose ASC or chemotherapy

	Chose chemotherapy	Chose ASC	*p*
Total (*n* = 139)	93 (66.9)	46 (33.1)	
Sex, *n* (%)			
Male	83 (89.2)	35 (76.1)	0.041
Female	10 (10.8)	11 (23.9)	
Laterality, *n* (%)			
Right	54 (58.1)	27 (58.7)	
Left	39 (41.9)	19 (41.3)	
			0.943
Age, mean (SD)			
	68.4 (6.36)	74.4 (7.35)	<0.001
Performance status, *n* (%)			
0	40 (43.0)	8 (17.4)	
1	48 (51.6)	32 (69.6)	
2	5 (5.4)	6 (13.0)	
			0.005
Histology, *n* (%)			
Epithelioid	63 (67.7)	36 (78.3)	
Sarcomatoid	15 (16.1)	6 (13.0)	
Biphasic	9 (9.7)	1 (2.2)	
Desmoplastic	2 (2.2)	1 (2.2)	
Not specified	4 (4.3)	2 (4.4)	
			0.540
IMIG tumour stage, *n* (%)			
IA	28 (30.1)	15 (32.6)	
IB	6 (6.5)	0	
II	7 (7.5)	3 (6.5)	
IIIA	0	0	
IIIB	30 (32.2)	13 (28.2)	
IV	10 (10.75)	7 (15.2)	
Not documented	12 (12.9)	8 (17.4)	
			0.543
Symptoms, *n* (%)			
Chest pain	38 (40.9)	22 (47.8)	0.435
Breathlessness	75 (80.7)	39 (84.8)	0.550
Cough	42 (45.1)	18 (39.1)	0.499
Systemic symptoms (sweats, weight loss, fatigue)	37 (39.8)	22 (47.8)	0.367
Blood tests, median (IQR)			
Haemoglobin, g/dL	138 (126-150)	132.5 (121-149)	0.263
Neutrophils, ×10^9^/L	5.73 (4.80-7.00)	5.84 (4.28-7.00)	0.729
Lymphocytes, ×10^9^/L	1.6 (1.15-2.15)	1.32 (1.00-1.94)	0.076
Albumin, g/L	35 (31-38)	34 (30-38)	0.456
Neutrophil Lymphocyte Ratio (NLR)	4.00 (2.73-5.38)	4.18 (3.09-6.38)	0.255

*ASC* active symptom control, *SD* standard deviation, *IQR* interquartile range

The group that chose ASC were older than those who accepted chemotherapy (mean age 74.4 vs 68.4, *p* < 0.001) and consisted of a higher proportion of females (23.9% vs 10.8%, *p* = 0.041). Additionally the group that chose ASC had poorer PS than the group that chose chemotherapy, with fewer PS 0 patients (17.4% vs 43.0%) and more PS 1 (69.6% vs 51.6%) and PS 2 patients (13.0% vs 5.4%, *p* = 0.005). There was no difference in laterality, histology, stage, symptoms or blood tests between the groups.

Of the 46 patients who chose ASC, all 46 were involved in initial discussions about first-line chemotherapy with a respiratory physician. 22/46 (47.8%) stated their decision to pursue ASC to the respiratory physician and consequently were not seen by an oncologist. 24/46 (52.2%) consulted with both respiratory physician and oncologist before deciding to pursue ASC.

In multivariable logistic regression analysis, the factors independently associated with choosing ASC were age (*p* < 0.001) and PS (*p* = 0.024).

Patients’ reasons for choosing ASC over first-line chemotherapy were documented in 15/46 (32.6%) cases. Reasons included concern that the benefits of chemotherapy did not justify the risk of side effects (5/15), a desire to prioritise quality of life in the context of no current symptoms (4/15), needle or hospital-phobia (3/15) and pursuit of alternative, experimental treatment in another country (1/15). Two (2/15) patients reported previous negative experiences with chemotherapy as their reason for choosing ASC. One of these patients had received chemotherapy for previous ovarian cancer, whilst the other had cared for his brother whilst he received chemotherapy for lung cancer.

### Secondary outcome – Survival

Of 139 MPM patients who were offered first-line chemotherapy, 122 (87.8%) died. Median follow up for surviving patients was 22.7 months (range 12.6 – 102.5 months). Survivors were censored on 26/06/17.

**Fig. 1:**
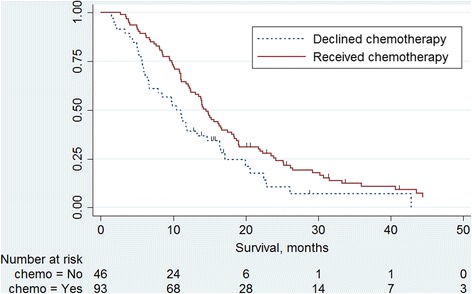
Kaplan Meier curves comparing survival in patients who chose ASC with those who chose chemotherapy

Median survival for all 139 patients was 13.5 months (interquartile range 7.9 – 20.0 months). Median survival in patients who received first-line chemotherapy was 14.5 months, compared with 10.8 months in patients who chose ASC having been offered chemotherapy (hazard ratio (HR) 0.62, 95% CI 0.42 – 0. 92, *p* = 0.016). Kaplan Meier survival curves are shown in Fig. 1.

In multivariable cox regression analysis, the factors independently associated with poor survival were PS of 2 (HR 2.61, 95% CI 1.17-5.84, *p* = 0.019), non-epithelioid histology (HR 1.79, 95% CI 1.16-2.76, *p* = 0.008), stage (HR 1.21, 95% CI 1.10-1.34, *p* < 0.001) and not receiving chemotherapy (HR 1.83, 95% CI 1.13-2.95, *p* = 0.014). There was a trend towards breathlessness at diagnosis being associated with poor survival (HR 1.83, 95% CI 0.99-3.36, *p* = 0.051).

## Discussion

This is the first study to report the characteristics of patients with MPM who were offered first-line chemotherapy but declined it in favour of ASC. In this prospective study of 139 patients, significant differences were observed between people who chose ASC and those who chose chemotherapy. This is an important finding in understanding attitudes to treatment in MPM, and potential factors affecting treatment decisions. Further research is needed to explore patient’s motivations for choosing ASC in greater depth, and the use of qualitative research methods could provide rich and informative data on this subject.

A strength of this study is the lack of missing data. Apart from one patient who moved out of the region, treatment choice and survival data was available for all patients. Data on tumour stage and histology was not recorded for a proportion of patients; but this is a phenomenon that has been observed nationally [2]. Overall, data completeness was high for this cohort, and this is likely to be a result of prospective data collection and rigorous database management.

Patients’ reasons for choosing ASC were collected retrospectively, and consequently were only available for one third of participants. Missing data is a recognised limitation of retrospective data collection, and may have introduced bias in this domain. It is acknowledged that the reasons for declining chemotherapy reported in this paper may not be representative of the whole group and that alternate reasons, not reported here, may also exist. Given the semi-qualitative nature of this outcome measure, results should be seen as hypothesis-generating, rather than conclusive. However, future studies would benefit from prospective collection of this information.

This study describes patients seen at a single UK centre and the results may not be generalizable. However, many of the findings from this study replicate other observational MPM studies. The male preponderance, the higher incidence of right-sided disease and the predominance of epithelioid sub-type are consistently reported, and are reproduced here [2, 3, 9]. Additionally the proportion of patients who were eligible for first-line chemotherapy, and the percentage of those people who went on to receive it were consistent with national rates, suggesting practice at our centre is similar to other centres in the UK [2, 9]. Finally, median survival of all MPM patients was comparable to previously reported survival times for MPM [2–4, 9]. The similarities between our cohort and national data suggest that patients in this study are representative of MPM patients in general. However, it is not known whether the characteristics of patients who choose ASC are the same in other centres. Further studies are needed to see whether similar results are observed elsewhere.

There was a difference in median survival of 3.7 months between patients who chose ASC and those who received first-line chemotherapy. It is likely that this survival difference represents more than just the biological effect of chemotherapy. Confounding due to the non-randomised, observational study design will have influenced survival, as patients who chose ASC had worse prognostic features, such as increased age and poorer PS. Additionally, unmeasurable factors may have contributed. For example, patients who chose ASC may have been less likely to seek medical help for other (treatable) medical problems, which could have impacted on their overall survival.

An important point to be considered in the interpretation of this study is the interaction between clinician and patient when discussing treatment options. It is possible that conversations regarding oncological treatments were more circumspect in older patients with poorer performance status, and that this influenced patient’s decisions. Clinician’s preferences, whether conscious or sub-conscious, implicit or explicit, could have affected the dynamic of the consultation and swayed patients towards ASC. An ethnographic, observational approach, or detailed conversation analysis, could be employed to investigate this possibility.

Where reasons for choosing ASC were given, they were varied and included concerns about chemotherapy side-effects and appreciation of the limited benefit offered by current first-line chemotherapy agents. Causal relationships cannot be assumed on the basis of this observational study. However, it may be that patients who were older and frailer had greater concerns about chemotherapy toxicity and were consequently more likely to choose ASC. Qualitative interviews around patients’ reasons for choosing ASC would be valuable in exploring this possibility in greater detail. Qualitative methods could also describe other potential reasons for choosing ASC or declining chemotherapy, and could reveal important factors in the decision-making process.

Several of the reasons given by patients for declining chemotherapy in this study have been reported in other studies of older adults with cancer, including concern about side effects, the wish to prioritise quality of life, and previous negative treatment experiences [17]. Additional reasons for declining cancer treatment that have been reported elsewhere include low mood and fear of becoming a burden on others or losing independence [17]. These factors were not reported in our cohort, but neither were they actively enquired about, due to retrospective data collection. Since none of the existing literature focuses specifically on people with mesothelioma, these are important areas that could be explored in future research in this patient group. Furthermore, financial considerations and transportation difficulties, have also been implicated in older patients’ decision-making around cancer treatments [17]. Whilst these issues may be less relevant in the UK where the NHS provides free universal healthcare, and hospital transportation is readily available, they may still be worth including in future prospective, qualitative research.

This study has highlighted the heterogeneity of patients with MPM, and identifies a specific sub-set of patients who choose not to have first-line chemotherapy. Further research is warranted to determine whether these findings are replicated in other centres and, indeed, other countries. Exploring patients’ attitudes to chemotherapy, and understanding the factors affecting the decision-making process could be the first step towards increasing treatment uptake, and potentially improving survival for MPM in the future.

## Conclusion

This is the first study to describe the characteristics of patients with mesothelioma who chose ASC over front-line chemotherapy. In this single-centre, UK-based study, participants who chose ASC were older, more likely to be female, and had worse performance status than those who accepted chemotherapy. Recognising these patients and understanding their motivations could improve communication and enhance the relationship between clinicians and patients.

## Appendix

Appendix 1 Inclusion and exclusion criteria for the Investigating Pleural Disease study.

## Inclusion criteria

1/. Undiagnosed/malignant pleural effusion or pleural thickening requiring investigation.

and

2/. Pleural aspiration or CT scan form part of the clinical plan.

Or

3/. Patients with a confirmed diagnosis of mesothelioma.

## Exclusion criteria

1/. Inability to give informed written consent.

2/. Pregnancy or lactation.

3/. Declines follow up to diagnosis or 12 months.

## Additional file

Additional file 1: Appendix 2 – Data collection form (DOCX 15 kb)

## Abbreviations

ASC: Active symptom control
CI: Confidence intervals
IQR: Interquartile range
MDT: Multidisciplinary team meeting
MPM: Malignant pleural mesothelioma
PS: Performance status
SD: Standard deviation
