# Supplementary material for: Exploring the characteristics of patients with mesothelioma who chose active symptom control over chemotherapy as first-line treatment: a prospective, observational, single centre study
Source: BMC Palliat Care. 2017 Dec 8;16:71. doi: 10.1186/s12904-017-0255-3 (PMC5723074; doi:10.1186/s12904-017-0255-3)
Supplement: Additional file 1: — Appendix 2 – Data collection form (DOCX 15 kb) [file 12904_2017_255_MOESM1_ESM.docx]

APPENDIX 2 – Data collection form

| Participant ID: |  | | | | | | | | | |  |  |  |  |  |  |  |
| --- | --- | --- | --- | --- | --- | --- | --- | --- | --- | --- | --- | --- | --- | --- | --- | --- | --- |
| Gender: | Male | | | | Female | | | | | |  |  |  |  |  |  |  |
| Age at diagnosis: |  | | | | | | | | | |  |  |  |  |  |  |  |
| Date of diagnosis: |  | | | | | | | | | |  |  |  |  |  |  |  |
| Co-morbidities | | | | | | | | | | |  |  |  |  |  |  |  |
| Cardiac | Yes | | | | No | | | | | |  |  |  |  |  |  |  |
| Renal | Yes | | | | No | | | | | |  |  |  |  |  |  |  |
| Other | Yes | | | | No | | | | | |  |  |  |  |  |  |  |
| What other? |  | | | | | | | | | |  |  |  |  |  |  |  |
| Symptoms at presentation | | | | | | | | | | |  |  |  |  |  |  |  |
| Chest pain | Yes | | | | No | | | | | |  |  |  |  |  |  |  |
| Breathlessness | Yes | | | | No | | | | | |  |  |  |  |  |  |  |
| Cough | Yes | | | | No | | | | | |  |  |  |  |  |  |  |
| Fatigue | Yes | | | | No | | | | | |  |  |  |  |  |  |  |
| Sweats | Yes | | | | No | | | | | |  |  |  |  |  |  |  |
| Weight loss | Yes | | | | No | | | | | |  |  |  |  |  |  |  |
| Performance status | 0 | | | 1 | | | 2 | | | 3 | | | | 4 | | | |
| Referred by: | GP | | | Resp team | | | Ward/inpatient | | | MAU | | | | Other hospital | | | |
| Biopsy method | VATS | | | LAT | | | CT guided | | | US guided | | | | Unknown | | | |
| Histological sub-type: | Epithelioid | | Sarcomatoid | | | | | Biphasic | | | | Desmoplastic | | | | NOS | |
| Laterality | Left | | | | | Right | | | | | | | Not stated | | | | |
| TNM stage |  | | | | | | | | | |  |  |  |  |  |  |  |
| IMIG stage | Ia | Ib | | | | II | | | III | | | | IV | | Not stated | | |
| Bloods at diagnosis | | | | | | | | | | |  |  |  |  |  |  |  |
| Haemoglobin |  | | | | | | | | | |  |  |  |  |  |  |  |
| Neutrophils |  | | | | | | | | | |  |  |  |  |  |  |  |
| Lymphocytes |  | | | | | | | | | |  |  |  |  |  |  |  |
| Neut/lymph ratio |  | | | | | | | | | |  |  |  |  |  |  |  |
| Albumin |  | | | | | | | | | |  |  |  |  |  |  |  |
| Eligible for chemo | Yes | | | | No | | | | | |  |  |  |  |  |  |  |
| Discussed with? | Pulmonologist | | | | Oncologist | | | | | |  |  |  |  |  |  |  |
| Treatment decision | Chemo | | | | ASC | | | | | |  |  |  |  |  |  |  |
| Reason for choosing ASC |  | | | | | | | | | | | | | | | |  |
| Outcome | Died | | | | Censored | | | | | |  |  |  |  |  |  |  |
| Survival (months) |  | | | | | | | | | |  |  |  |  |  |  |  |
